# Family Economic Hardship and Non-Suicidal Self-Injury Among Chinese Adolescents: Relative Deprivation as a Mediator and Self-Esteem as a Moderator

**DOI:** 10.3390/bs14121234

**Published:** 2024-12-22

**Authors:** Xiaoyan Liao, Huahua Wang, Xingcan Ni, Chengfu Yu

**Affiliations:** 1 Department of Psychology, Research Center of Adolescent Psychology and Behavior, School of Education, Guangzhou University, Guangzhou 510006, China2112308033@e.gzhu.edu.cn (X.N.); 2 School of Psychology, South China Normal University, Guangzhou 510631, China

**Keywords:** adolescent, non-suicidal self-injury (NSSI), family economic hardship, relative deprivation, self-esteem

## Abstract

The literature provides empirical evidence that family economic hardship can increase the likelihood of adolescents engaging in non-suicidal self-injury (NSSI). However, the mechanisms underlying this relationship remain unclear. Guided by the risky families model, this study aimed to investigate whether relative deprivation mediates the link between family economic hardship and adolescent NSSI, and whether self-esteem moderates this indirect link. A combined 673 adolescents (45.9% female; *M*_age_ = 12.81 years) completed measures of family economic hardship, relative deprivation, self-esteem, and NSSI. The results verified that relative deprivation mediated the positive link between family economic hardship and NSSI. Additionally, self-esteem moderated the effect of family economic hardship on relative deprivation. Adolescents possessing high self-esteem reported lower relative deprivation compared to those possessing low self-esteem, regardless of their level of family economic hardship. Notably, the mitigating effect of high self-esteem diminished with high family economic hardship. These findings provide deeper insights into the mechanisms by which family economic hardship affect adolescent NSSI, and have practical implications for prevention and intervention strategies targeting this behavior.

## 1. Introduction

Non-suicidal self-injury (NSSI) refers to intentional, self-inflicted harm to one’s body tissue without suicidal intent, commonly including cutting, scratching, banging, biting, or burning [1]. NSSI has become an alarming psychological well-being issue among adolescents [2], with its incidence rising annually. A recent study indicated that the six-month incidence of NSSI among teenagers in China is approximately 16.4% [3]. It not only seriously affects adolescents’ physical health but also increases the likelihood of future suicide attempts and suicide, imposing a substantial burden on society and families [4]. With the increasing prevalence and harmful outcomes of NSSI, identifying risk and protective factors is vital for formulating efficacious prevention and intervention strategies.

Numerous factors contribute to adolescent NSSI. Family stressors have long been recognized as a major risk factor for adolescent NSSI [5]. Family economic hardship, a crucial aspect of familial stressors, involves insufficiency in the family’s financial circumstances for fulfilling its needs [6]. According to the experiential avoidance model, children and adolescents from economically disadvantaged families who experience the adversity and limited supportive resources are more prone to experiencing uncomfortable or distressing emotions [7]. To avoid or mitigate these emotions, adolescents may resort to self-injury as an ineffective coping strategy [7]. During adolescence, a period marked by increasing material needs and self-identity formation, individuals become more sensitive to economic hardships, making them more vulnerable to family financial struggles [8]. Adolescents whose families are experiencing economic hardship often face material constraints, such as cramped living conditions and limited living space, as well as non-material resource challenges, including strained family relationships and limited parental care; these situations can hinder their growth and mental health [9]. Studies have revealed that family economic hardship can increase the risk of NSSI in adolescents [10,11,12]. Markedly, a recent longitudinal study found that subjective socioeconomic status negatively predicted NSSI frequency [13].

It is worth noting that previous studies in Western countries have found that the association between family economic hardship and children’s mental and physical health is not as strong as expected [14,15,16]. By contrast, research in China and other Asian countries demonstrate a significant relationship between family economic hardship and the mental health of children and adolescents [17,18]. Moreover, China’s ongoing reforms and sophisticated development, which have resulted in a widening of economic disparities, have exacerbated the challenges related to social mobility and accentuated the social hierarchy, intensifying the impact of family economic hardship on adolescents’ NSSI. Therefore, it is crucial to explore the mechanisms through which family economic hardship affects NSSI among Chinese adolescents. Previous research has primarily examined the indirect impact of family economic hardship on adolescents’ NSSI via parental influence [19], overlooking its direct impact on their subjective experience—a crucial direction ripe for further exploration. Adolescents’ subjective experiences, like relative deprivation and self-esteem, constitute vital components of their physical and mental well-being. Thus, this study aims to explore the mechanisms through which family economic hardships impact NSSI among adolescents’ living in China, focusing on subjective experiences (such as relative deprivation). By examining adolescents’ subjective experiences under family economic hardships, we can better understand their psychological reactions and behavioral patterns. Such insights are crucial for designing effective interventions to reduce NSSI risk.

### 1.1. The Mediating Effect of Relative Deprivation

Relative deprivation arises from social comparisons, where an individual or group subjectively perceives themselves as disadvantaged relative to a reference group. This is often accompanied by emotions such as anger, resentment, and frustration [20]. During adolescence, the increased peer interaction creates numerous opportunities for adolescents to engage in social comparisons [21]. These comparisons extend beyond academic achievements to include family economic situations. When comparing their family’s economic situations with those of their peers, adolescents from economically disadvantaged families may perceive themselves as being at a disadvantage, consider themselves as having a lower social status, and consistently view this situation as unfair, triggering a heightened sense of relative deprivation [22]. Recent studies confirm the positive association between family economic hardship and adolescent relative deprivation [19,23]. Adolescents with a high sense of relative deprivation often focus on their own disadvantages, exacerbating the fear of failure [24]; this leads to increased rumination and experiential avoidance [25], ultimately heightening their vulnerability to NSSI [26]. Empirical studies also revealed that relative deprivation predicts NSSI [26,27]. The experiential avoidance model suggests that individuals who encounter family economic disadvantage often experience relative deprivation accompanied by negative emotions [7]. Without effective emotion regulation strategies, they may turn to NSSI as a temporary means of alleviating negative feelings [7]. Therefore, relative deprivation may play a mediating role in the link between family economic hardship and NSSI. Despite the absence of direct evidence for this mediating role, existing research provides circumstantial evidence. For example, Yu et al. [28] found that Chinese college students with lower subjective social class experienced higher relative deprivation, which contributed to greater interpersonal distrust. Similarly, another study revealed that relative deprivation exerted a mediating effect between subjective socioeconomic status and depression [29]. These findings underscore the importance of exploring how relative deprivation mediates the link between family economic hardship and NSSI in adolescents.

### 1.2. The Moderating Effect of Self-Esteem

While family economic hardship may influence NSSI among adolescents via relative deprivation, not all adolescents are equally affected. Previous research indicates that family economic conditions interact with individual characteristics such as self-esteem to shape adolescent development [30]. Self-esteem, defined as how a person positively perceives and evaluates themselves [31], is a strong indicator of adolescent physical and mental health [32,33]. The buffering model of self-esteem posits that individuals possessing low self-esteem experience greater distress from adversity, whereas those with high self-esteem are better shielded against stressors or difficulties [34]. Studies consistently show that self-esteem buffers the link stressors and negative outcomes [35,36]. In addition, self-esteem may influence how adolescents process social comparisons induced by family economic hardship. Individuals with low self-esteem tend to focus on the negative aspects of social comparison, leading to intensified feelings of inferiority, psychological disadvantage, and increased relative deprivation [37,38]. Conversely, those possessing high self-esteem are more likely to view such comparisons as challenges rather than threats. They actively employ self-regulation strategies and make greater efforts to resolve them [38,39]. This evidence suggests that high self-esteem may mitigate the adverse impacts of family economic hardship on relative deprivation. Adolescents with low self-esteem who perceive their family as being economically disadvantaged compared to others may experience intense feelings of psychological disadvantage and increased relative deprivation. However, few studies have examined how self-esteem moderates the correlation between family economic hardship and relative deprivation.

**Figure 1:**
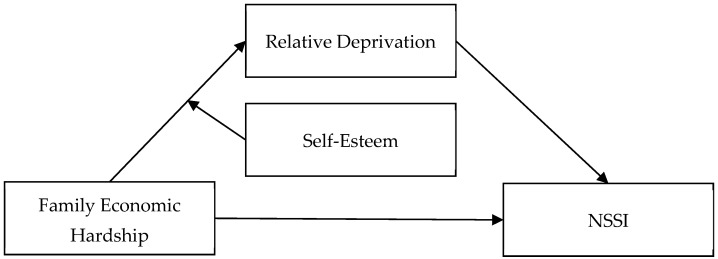
Hypothetical moderated mediation model. Note: NSSI, non-suicidal self-injury.

This study explored how family economic hardship is linked to NSSI among adolescents in China through a moderated mediation model. After reviewing the above research, we hypothesized the following (see Figure 1):

**H1.**  *The positive link between family economic hardship and NSSI is mediated by relative deprivation.*

**H2.**  *Self-esteem moderates the link between family economic hardship and relative deprivation.*

## 2. Materials and Methods

### 2.1. Participants

In this study, the sample size was computed utilizing G*Power 3.1.9.7 based on the statistical parameters obtained from previous studies [40,41]. Interaction *R^2^* values were estimated to range between 0.03 and 0.09. Statistical computations showed that a cohort of 119–353 participants would yield a statistical power of at least 90% (two-tailed α = 0.05).

Participants were selected from two middle schools in Dongguan City, Guangdong Province, South China. Using a cluster sample technique, the schools were chosen from a group of general public middle schools, stratified by class as the sampling unit. The selection ensured an equal mix of male/female and urban/rural students. Three additional participants were disqualified from the study due to their submission of incomplete or careless responses on over 30% of the survey items. Ultimately, 673 students (45.9% female; *n* = 309) aged 11–15 years (*M*_age_ = 12.81 years; *SD* = 0.48 years) diligently participated in the study. The majority of survey respondents (91.3%) lived in households headed by two parents. Furthermore, only 10.4% of mothers and 12.8% of fathers had completed at least a tertiary education, and 33.0% of the families reported monthly incomes of RMB 5000 or higher.

### 2.2. Procedure

All of the procedures were approved by the Ethics in Human Research Committee of the School of Education at Guangzhou University. Informed consent was obtained from both the adolescents and their parents prior to participation. Psychology teachers, who had undergone rigorous training, and graduate psychology students distributed self-reported questionnaires in classroom settings. The questionnaire took approximately 45 min to complete and was collected by the interviewers upon completion. Students were informed that participation was entirely voluntary and they could decline without facing any negative consequences.

Throughout the survey, interviewers maintained open communication, encouraging participants to ask any questions or express any concerns. They constantly monitored the participants’ reactions and emotional states during the filling process. To alleviate any adverse experiences from disclosing painful experiences, such as NSSI, participants received a pen as a comfort gesture at the end of the survey. Contact information for the school’s psychological counseling center was also provided to ensure access to professional psychological support and intervention if needed.

### 2.3. Measures

#### 2.3.1. Family Economic Hardship

The Family Economic Hardship Questionnaire, adapted from the Responses to Stress Questionnaire [42], was utilized to evaluate family economic hardship [43]. The previous literature has shown the adequate reliability and validity of the original and adapted measure [44,45]. The scale contains four items (e.g., “My family does not have enough money to buy new clothes”). Answers to the items were given on a 4-point Likert scale ranging from 1 (“never”) to 4 (“always”), with higher scores indicating more severe family economic hardship. In this research, Cronbach’s α was calculated to be 0.84.

#### 2.3.2. Relative Deprivation

The Relative Deprivation Questionnaire was employed to measure adolescents’ relative deprivation [46]. This scale has shown adequate reliability and validity in Chinese adolescents [47,48]. The scale was comprised of four items (e.g., “Compared to the people around me, I’m at a disadvantage in every aspect of my life, my studies, etc.”), each of which was rated on a 6-point Likert scale ranging from 1 (“strongly disagree”) to 6 (“strongly agree”). Greater scores are indicative of increased levels of relative deprivation. In this research, Cronbach’s α was calculated to be 0.68.

#### 2.3.3. Non-Suicidal Self-Injury

The Chinese version of the NSSI Questionnaire, adapted from the Responses to Stress Questionnaire [49], was utilized to evaluate NSSI [50]. This questionnaire asked participants to recall and report the frequency of six types of self-injury behaviors that do not include suicidal intent (e.g., self-directed cutting, engraving on the skin with a sharp object, scratching the skin, pulling hair, biting, and rubbing the skin so hard that it bleeds) in the past six months. These NSSI behaviors are relatively common among adolescents, and the relevant measure has shown the adequate reliability and validity of this measure among Chinese adolescents [1,51]. The responses to the items were measured on a 6-point Likert scale ranging from 1 (“never”) to 6 (“several times a week”). The items’ mean score was computed, with a higher score indicating more severe NSSI. In this research, Cronbach’s α was calculated to be 0.70.

#### 2.3.4. Self-Esteem

Self-esteem was evaluated through the utilization of the Rosenberg Self-Esteem Scale, which has ten items (e.g., “Generally speaking, I am satisfied with myself”) rated on a 4-point scale ranging from 1 (“strongly disagree”) to 4 (“strongly agree”) [52]. Increased scores suggested higher levels of self-esteem. Previous studies have demonstrated good levels of reliability and validity for this scale among Chinese adolescents [53,54]. Cronbach’s α was calculated to be 0.81 in this study.

#### 2.3.5. Control Variable

Age and gender were control variables because previous studies found that they were closely related to the observed variables [55,56].

### 2.4. Data Analyses

The data were analyzed using SPSS 26.0 (SPSS; IBM, Armonk, NY, USA). The missing values were addressed using the average interpolation method. Prior to conducting hypothesis testing, we assessed data normality through skewness and kurtosis tests. The results showed normal distributions for family economic hardship, relative deprivation, and self-esteem, with skewness and kurtosis remaining within acceptable ranges (skewness < |2.0| and kurtosis < |7.0|). However, the NSSI demonstrated a non-normal distribution (skewness > 2.0; kurtosis > 7.0) [57,58]. Therefore, mediation and moderation were analyzed using bootstrapping with 5000 samples. This method yields bias-corrected, accelerated 95% CIs without assuming normality [59,60]. The Harman’s single-factor test showed that the variance for both rotated and unrotated primary factors was below 40%, indicating no significant common method bias in the study [61]. In addition, as all predictors had a VIF below 2, multicollinearity did not exist in the study [62].

Initially, the main variables were analyzed to calculate the means, standard deviations, and bivariate correlations. Subsequently, the mediating effect of relative deprivation was assessed through macro PROCESS Model 4 [63]. Last, the assessment of the moderated mediation model was conducted using macro PROCESS Model 7. All continuous measures were standardized before conducting the analyses.

## 3. Results

### 3.1. Descriptive Statistics

The study variables’ descriptive statistics and Pearson’s correlations are presented in Table 1. As expected, family economic hardship, relative deprivation, and NSSI were positively correlated, while each of these variables were negatively associated with self-esteem. Additionally, the means values for family economic hardship, relative deprivation, self-esteem, and NSSI aligned with the findings from previous research [45,51,53,64].

### 3.2. Testing for Mediation Effect

Next, a mediation analysis was conducted to explore the influence of relative deprivation in the link between family economic hardship and NSSI. As shown in Table 2, when controlling for age and gender, family economic hardship was positively linked to both relative deprivation (*β* = 0.20, *p* < 0.001) and NSSI (*β* = 0.11, *p* < 0.01). Relative deprivation was also positively linked to NSSI (*β* = 0.10, *p* < 0.01). There was a significant direct link between family economic hardship and NSSI (*β* = 0.11, *SE* = 0.04, *p* < 0.01). Bootstrapping analyses (*n* = 5000) revealed a significant indirect effect of family economic hardship → relative deprivation → NSSI (indirect effect = 0.02, *SE* = 0.01, and 95% CI [0.01, 0.04]), accounting for 15.38% of the total effect (see Table 3). Therefore, H1 was supported.

### 3.3. Testing for Moderated Mediation

The results of the test of the moderated mediation model are shown in Table 4. Controlling for age and gender, Model 1 showed that family economic hardship had a significantly positive effect on relative deprivation (*β* = 0.21, *p* < 0.001), self-esteem had a significantly negative effect on relative deprivation (*β* = −0.14, *p* < 0.001), and the interplay between family economic hardship and self-esteem was significantly associated with an increase in relative deprivation (*β* = 0.07, *p* < 0.05). In Model 2, both family economic hardship and relative deprivation had a significant and detrimental impact on the likelihood of engaging in NSSI (*β* = 0.10, *p* < 0.05).

**Figure 2:**
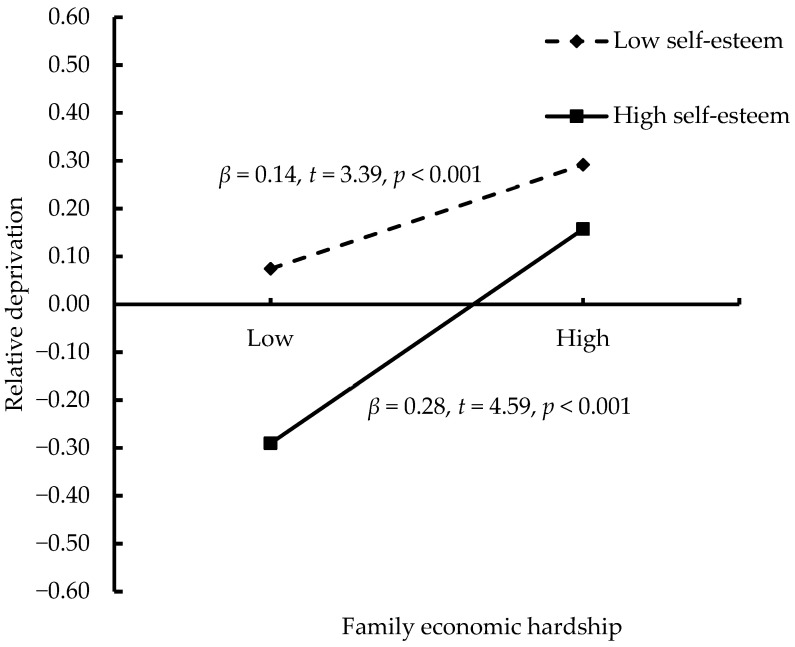
Interactive effect of family economic hardship and self-esteem on relative deprivation.

To further explore the interaction effect, we performed simple slopes tests. The results (see Figure 2) showed that adolescents possessing high self-esteem experienced lower relative deprivation compared to those possessing low self-esteem, regardless of their level of family economic hardship. However, for adolescents possessing high self-esteem, the link between family economic hardship and relative deprivation was stronger (*β* = 0.28 and *SE* = 0.06; *p* < 0.001) than for those possessing low self-esteem (*β* = 0.14 and *SE* = 0.04; *p* < 0.001).

The indirect effect of family economic hardship → relative deprivation → NSSI, moderated by self-esteem, was analyzed through bootstrapped confidence intervals (see Table 5). The result revealed that the mediating effects of relative deprivation in the pathway from family economic hardship to NSSI were stronger in adolescents with a high self-esteem (*β* = 0.29 and *SE* = 0.06; 95% CI [0.16, 0.40]) than in those with low self-esteem (*β* = 0.14 and *SE* = 0.04; 95% CI [0.06, 0.22]). Therefore, the link between family economic hardship and relative deprivation was significantly moderated by self-esteem, supporting H2. Specifically, high self-esteem had a greater mitigating effect on the link than low self-esteem.

## 4. Discussion

Using a moderated mediation model, this study investigated the psychological mechanisms underlying the link between family economic hardship and NSSI among adolescents. The findings revealed that adolescent NSSI was positively and strongly associated with family economic hardship, a relationship partially mediated by relative deprivation. Furthermore, self-esteem moderated role in the link between family economic hardship and relative deprivation. These findings highlight the important roles of relative deprivation and self-esteem in the impact of family economic hardship on adolescent NSSI.

Consistent with previous studies, family economic hardship was linked to an increased risk of NSSI in adolescence [10,12], supporting the experiential avoidance model [7]. Family economic hardship often prevents the fulfillment of adolescents’ material needs, creating stress and negative feelings when there is a mismatch between their needs and available resources [65]. Additionally, family economic hardship can impair the parenting quality, as parents may prioritize material concerns, reducing parental social support and emotional attention for adolescents [9]. This lack of effective emotional warmth and timely guidance from their parents may lead adolescents to adopt maladaptive strategies when faced with difficulties. Thus, under family economic hardship, adolescents are more prone to experiencing stress and a depletion of their psychological resources, impeding the development of positive emotions, behaviors, and cognition in adolescents [66]. Adolescents are also more likely to adopt maladaptive strategies to alleviate negative feelings (e.g., by engaging in NSSI) [13,67].

### 4.1. The Mediating Effect of Relative Deprivation

As hypothesized, relative deprivation significantly mediated the relationship between family economic hardship and NSSI. This suggests that family economic hardship influences NSSI both directly and indirectly by exacerbating relative deprivation, thereby increasing the likelihood of NSSI and, consequently, supporting the experiential avoidance model. This result extends prior studies that mainly explored the role of the parents’ relationship, the parent–child relationship, and other factors, neglecting adolescents’ subjective attitudes (e.g., relative deprivation) [68,69]. These findings also provide support for relative deprivation theory [20], which suggests that adolescents from economically disadvantaged families face limited material and social resources, and perceive more relative deprivation through social comparison. Adolescents with a high relative deprivation often focus on their own disadvantages, which leads to emotional distress and maladjustment [70,71]. The sense of relative deprivation is not only accompanied by emotional distress but also affects individuals’ physiology. Moreover, the cascade of negative emotions induced by a sense of relative deprivation may increase the allostatic load, thereby harming the sustained overactivity of the hypothalamic–pituitary–adrenal axis system [72,73]. These changes in biochemical processes may increase the susceptibility to NSSI [74,75]. NSSI may serve as a coping strategy for individuals to alleviate the negative feelings caused by relative deprivation; the intense physiological reactions triggered by NSSI effectively divert attention away from negative emotions, enabling a temporary escape and relief. Considering the function of NSSI in regulating negative affect, adolescents tend to adopt the behavior in order to temporarily relieve emotional pain [76]. Noteworthy, the mediation effect could only partially explain the association between family economic hardship and NSSI, and only accounted for a modest portion of the total effect. This underscores the possibility of additional pathways through which family economic hardship affects NSSI. Consequently, future research efforts should delve deeper into the intricate ways in which family economic hardship affects NSSI to gain a more comprehensive understanding of this phenomenon.

### 4.2. The Moderating Effect of Self-Esteem

Consistent with H2, self-esteem moderated the link between family economic hardship and relative deprivation. Specifically, adolescents with a high self-esteem experienced lower relative deprivation than those possessing a low self-esteem regardless of their level of family economic hardship. Individuals possessing a low self-esteem are prone to self-blame, and consequently focus on negative emotions about the self during adversity [77]. This could explain why they experience more relative deprivation when their family is undergoing economic hardship. Conversely, individuals possessing a high self-esteem tend to use more effective coping styles and have greater resilience; therefore, they are less likely to experience negative emotional states when they encounter adversity [78]. Thus, adolescents with a high self-esteem experience less relative deprivation linked to family economic hardship. This pattern supports the buffering model of self-esteem [34], which posits that individuals with a high self-esteem, when engaging in social comparisons, tend to select more favorable goals and dimensions for evaluation, thereby bolstering their self-identity and mitigating negative emotions [34]. These results are congruent with prior research. For example, Wright et al. [79] discovered that self-esteem lowered perceived stress and internalizing symptoms. Similarly, Wang et al. [36] found that adolescents possessing high self-esteem experienced lower depression than those possessing low self-esteem when exposed to parental phubbing, a phenomenon where parents ignore their child or are distracted by smartphones during parent–child interactions.

Notably, the effect of family economic hardship on relative deprivation was stronger among adolescents possessing a high self-esteem than those possessing a low self-esteem. This suggests that the mitigating effect of high self-esteem diminishes with high family economic hardship. Beyond its inherent negative impacts, family economic hardship also weakens the positive influence of protective factors. Adolescents with a strong self-esteem have higher aspirations [80,81], which may lead to discrepancies between their expectations and reality in economically challenging situations. Such discrepancies heighten the sensitivity to relative deprivation. Recent studies have also discovered that self-esteem has a limited ability to buffer the negative effects of stressful events [82,83]. Finally, previous research has also suggested that different kinds of self-esteem affect individual cognition [84]. This study focused on explicit self-esteem, measured using the Rosenberg Self-Esteem Scale, for evaluation purposes. Future research can systematically compare the specific roles of different types of self-esteem (e.g., explicit and implicit self-esteem) with respect to their influence on the effect of family economic hardship on relative deprivation.

### 4.3. Limitations and Future Research

The current study has several limitations. First, its cross-sectional design prevents causal inferences. Longitudinal and experimental studies are needed to confirm the effects of family economic hardship on adolescents’ NSSI. Second, all of the data were self-reported by the adolescents. Future studies should incorporate multi-agent evaluation methods for data collection. Third, the indirect effect of relative deprivation was relatively low, potentially attributable to the influence of other variables, including parental factors. Adolescents may experience high relative deprivation and low self-esteem due to a poor parent–child relationship and/or the parental relationship [19]. Future research should examine the common effect of multiple factors on relative deprivation and aim to reveal their unique contributions. Last, the generalizability of this study’s findings may be limited because the sample only included adolescents from two schools in Guangdong Province, a relatively developed region in China. Further research is necessary to ascertain the applicability of our findings to adolescents in various regions or cultural settings. In the future, sample characteristics could be expanded to improve the applicability of the results.

### 4.4. Implications

This study offers crucial implications for the prevention and treatment of adolescent NSSI. First, it highlights the harmful effects of family economic hardship on NSSI. To prevent maladaptive behaviors such as NSSI in adolescents, the government and society should provide the necessary materials, including the establishment of scholarship programs and tuition reductions, to mitigate the adverse impacts of family financial hardship. Second, our study uncovered that the connection between family economic hardship and NSSI was mediated by relative deprivation. Based on previous studies, we should not only provide a positive family environment for adolescents but also pay attention to the subjective experiences of adolescents in economically disadvantaged families. Thus, it is essential to provide timely and effective psychological support. For example, cognitive training could effectively intervene and alleviate relative deprivation over a short period of time. Specifically, adolescents should learn reasonable social comparisons, change negative perceptions, and adopt emotional regulation strategies to mitigate the risk of relative deprivation. Last, as found in this study, self-esteem can significantly influence the indirect relationship between family economic hardship and NSSI. Parents and educators play a crucial role in assisting adolescents in cultivating optimistic self-assessments and well-defined self-perceptions. For example, parents and educators assist adolescents in developing a positive self-image and self-worth through encouragement and affirmation. Additionally, schools can offer mental health programs, such as dance–movement therapy, to enhance adolescents’ self-esteem [85].

## 5. Conclusions

This study established a moderated mediation model with a group of Chinese junior high school students, providing an elaborate understanding of how family economic hardship is related to adolescent NSSI. The results show that relative deprivation can partially explain the positive correlation between economic hardship and NSSI. Furthermore, self-esteem moderated the mediating process. This study offers empirical support for the link between family economic hardship and NSSI, which can contribute to developing effective psychological interventions to reduce NSSI among adolescents.

## Figures and Tables

**Table 1:** Descriptive statistics and correlations for the variables.

Variable	*M*	*SD*	1	2	3	4	5	6
1. Age	12.81	0.48	1.00					
2. Gender	-	-	0.06	1.00				
3. FEH	1.33	0.57	0.03	0.03	1.00			
4. RD	2.28	0.97	0.01	0.08 *	0.20 ***	1.00		
5. SE	2.96	0.52	−0.03	0.02	−0.20 ***	−0.17 ***	1.00	
6. NSSI	1.07	0.23	−0.01	−0.06	0.13 **	0.12 **	−0.13 **	1.00

Note: FEH, family economic hardship; RD, relative deprivation; SE, self-esteem; NSSI, non-suicidal self-injury. Gender was coded as 1 = male and 0 = female. * *p* < 0.05, ** *p* < 0.01, and *** *p* < 0.001.

**Table 2:** The mediation effect of family economic hardship on NSSI.

Variables	Model 1 (RD)				Model 2 (NSSI)			
	*β*	*SE*	*t*	95% CI	*β*	*SE*	*t*	95% CI
Age	0.00	0.04	−0.04	[−0.08, 0.07]	−0.01	0.04	−0.25	[−0.08, 0.07]
Gender	0.15	0.08	1.93	[0.00, 0.30]	−0.14	0.08	−1.77	[−0.29, 0.02]
FEH	0.20	0.04	5.21 ***	[0.12, 0.27]	0.11	0.04	2.77 **	[0.03, 0.18]
RD					0.10	0.04	2.67 **	[0.03, 0.18]
*R^2^*	0.05				0.03			
*F*	10.51 ***				5.17 ***			

Note: FEH, family economic hardship; RD, relative deprivation; NSSI, non-suicidal self-injury. ** *p* < 0.01, *** *p* < 0.001.

**Table 3:** Total, direct, and indirect effects of family economic hardship on NSSI.

	Effect	Boot SE	Boot LLCI	Boot ULCI	Ratio
Indirect effect	0.02	0.01	0.01	0.04	15.38%
Direct effect	0.11	0.04	0.03	0.18	84.62%
Total effect	0.13	0.04	0.05	0.20	100%

Note: NSSI, non-suicidal self-injury.

**Table 4:** Testing the moderated mediation effect of family economic hardship on NSSI.

Variables	Model 1 (RD)				Model 2 (NSSI)			
	*β*	*SE*	*t*	95% CI	*β*	*SE*	*t*	95% CI
Age	−0.01	0.04	−0.28	[−0.08, 0.06]	−0.01	0.04	−0.25	[−0.08, 0.07]
Gender	0.15	0.08	2.01 *	[0.00, 0.30]	−0.14	0.08	−1.77	[−0.29, 0.02]
FEH	0.21	0.04	5.02 ***	[0.13, 0.29]	0.11	0.04	2.77 **	[0.03, 0.18]
RD					0.10	0.04	2.67 **	[0.03, 0.18]
SE	−0.14	0.04	−3.68 ***	[−0.21, −0.07]				
FEH × SE	0.07	0.03	2.34 *	[0.01, 0.13]				
*R^2^*	0.07				0.03			
*F*	10.28 ***				5.17 ***			

Note: FEH, family economic hardship; RD, relative deprivation; SE, self-esteem; NSSI, non-suicidal self-injury. * *p* < 0.05, ** *p* < 0.01, and *** *p* < 0.001.

**Table 5:** Bootstrapping analysis of the moderated mediation effect.

Levels of Self-Esteem	Effect Size	Boot SE	95% CI
1. Low (M−SD)	0.14	0.04	[0.06, 0.22]
2. Med (M)	0.21	0.04	[0.13, 0.29]
3. High (M+SD)	0.29	0.06	[0.16, 0.40]

## Data Availability

The datasets used and analyzed during the current study are available from the corresponding author on reasonable request.
